# Integrated analysis of N-glycosylation and Alzheimer’s disease: identifying key biomarkers and mechanisms

**DOI:** 10.3389/fnagi.2025.1597511

**Published:** 2025-11-13

**Authors:** Hao Zhang, WenJun Chen, ShuYou Yuan, Bo Cai, ShaoXiang Ding, HongXia Bao, JunKai Sun, Wei Lu, HaoGang Zhu

**Affiliations:** 1 Geriatrics Center, Wuxi Second Geriatric Hospital, Wuxi, China; 2 Department of Neurology, Wuxi Second Geriatric Hospital, Wuxi, China; 3 Department of Laboratory, Wuxi Second Geriatric Hospital, Wuxi, China; 4 Department of Pathology, Wuxi Second Geriatric Hospital, Wuxi, China; 5 Department of Interventional Radiology, Wuxi No. 5 People’s Hospital, Wuxi, China

**Keywords:** Alzheimer’s disease, N-glycosylation, diagnostic biomarkers, machine learning, transcription factors

## Abstract

**Background:**

Alzheimer’s disease (AD) is the most prevalent cause of dementia in the elderly, imposing a significant societal burden. Current therapeutic approaches primarily address symptoms, underscoring the critical need to elucidate its pathogenesis and identify robust early biomarkers. N-glycosylation, a critical post-translational modification, is dysregulated in neurodegenerative disorders, yet its role in AD and diagnostic potential remain underexplored.

**Objective:**

This investigation aimed to characterize the interplay between N-glycosylation and AD through multi-dimensional bioinformatics analysis, identify core differentially expressed genes (DEGs) associated with this crosstalk, and evaluate their diagnostic efficacy in early AD detection.

**Methods:**

A bibliometric analysis of Web of Science literature spanning 2001–2025 was performed using VOSviewer, CiteSpace, and R. Transcriptomic data were analyzed with LIMMA to identify DEGs. Feature prioritization and molecular interaction decoding were achieved through Lasso, Random Forest, XGBoost, and SHAP analysis.

**Results:**

Bibliometric analysis highlighted a shift toward granular molecular mechanisms, with “bisecting GlcNAc” and “GNT-III (MGAT3)” emerging as key research topics. Differential expression profiling identified 6,845 DEGs, including TMEM59, MLEC, and MAX. Machine learning algorithms consistently prioritized these three genes as core N-glycosylation-related biomarkers, alongside APP as a key associated molecule. Among transcription factors, MAX was identified as a central regulator, with a subset of 8 factors (including MAX and BRD9) pinpointed as critical modulators of N-glycosylation and glial activation in AD. Diagnostic models demonstrated strong performance: logistic regression achieved an AUC of 0.947 with MAX, APP, and MLEC; Random Forest and XGBoost attained perfect AUC = 1.0 in primary analyses; and a clinical nomogram integrating core genes yielded an AUC of 0.899. SHAP analysis confirmed MAX, APP, MLEC, and TMEM59 as top predictors, revealing significant positive interactions between MLEC and TMEM59 (*p* = 0.00019) and a negative interaction between MAX and MGAT3 (*p* = 0.0288). Notably, MAX alone served as a impactful single-gene biomarker, with AUC values ranging from 0.644 to 0.898 across external validation.

**Conclusion:**

MAX, MLEC, and TMEM59 represent key N-glycosylation-linked diagnostic biomarkers for AD. This integrative framework provides novel insights into AD pathogenesis and lays the foundation for personalized diagnostic tools and therapies, warranting experimental validation.

## 1 Introduction

Alzheimer’s disease (AD) is a progressive neurodegenerative disorder and the leading cause of dementia in the elderly population. It is characterized by a gradual decline in cognitive function and impairment of daily living activities, imposing a significant burden on affected individuals, their families, and society (Kawaharada et al., 2019). The rising prevalence of AD aligns with global demographic shifts toward an aging population, making it a critical public health priority. Projections indicate that by 2,050, dementia incidence will double in Europe and triple globally, with AD accounting for the majority of cases (You, 2025). Beyond its direct impact on patients, AD also affects caregivers, who often face psychological, physical, and economic strain, as well as healthcare systems, which must address the growing demand for long-term care and support services (Fang et al., 2024). Despite extensive research and resource allocation, current therapeutic approaches—both pharmacological (e.g., cholinesterase inhibitors and NMDA receptor antagonists) and non-pharmacological—remain largely symptomatic and do not halt or reverse disease progression (Kaye et al., 2024). This limitation underscores the urgent need to elucidate AD pathogenesis and identify reliable biomarkers for early diagnosis and effective management.

Significant progress has been made in characterizing the molecular hallmarks of AD, including amyloid-β plaques and neurofibrillary tangles composed of hyperphosphorylated tau protein (Huang and Jiang, 2009). However, emerging research highlights the complexity of AD, implicating a diverse array of genetic, epigenetic, metabolic, and environmental factors that contribute to its heterogeneity and clinical variability (Migliore and Coppedè, 2022). Among these, post-translational protein modifications, particularly N-glycosylation, have garnered increasing attention due to their critical roles in protein folding, trafficking, and cell signaling (Hou et al., 2023). N-glycosylation, a highly conserved enzymatic process involving the attachment of oligosaccharides to asparagine residues, is essential for proper protein conformation and function (Dünser and Schoberer, 2025). Dysregulation of N-glycosylation pathways has been implicated in the pathogenesis of various diseases, including neurodegenerative disorders, suggesting that alterations in glycosylation may contribute to protein aggregation, synaptic dysfunction, and neuroinflammation in AD (Xu et al., 2025a).

Despite these insights, the precise role of N-glycosylation in AD pathogenesis remains incompletely understood. While studies have documented the importance of N-glycans in neuronal protein homeostasis and synaptic plasticity, direct evidence linking specific N-glycosylation-related genes to AD onset and progression is limited (Lee et al., 2017). Furthermore, few investigations have systematically evaluated the diagnostic utility of N-glycosylation profiles or associated genetic markers in AD, in contrast to established biomarkers such as amyloid-β, tau, and neuroimaging modalities. This knowledge gap represents a critical obstacle to the development of novel, non-invasive diagnostic strategies for early AD detection and risk stratification (Khan, 2018). Additionally, existing studies often rely on single-omic or unidimensional analytic approaches, lacking the integrative perspective necessary to disentangle the complex molecular networks underlying AD (Xu et al., 2025b).

To address these challenges, this study integrates multi-dimensional bioinformatic and statistical methods to systematically explore the intersection of N-glycosylation and AD. By combining bibliometric analysis, gene expression profiling, and machine learning, this work seeks to overcome previous limitations. Bibliometric approaches map thematic evolution and identify research frontiers within the N-glycosylation–AD field, while differential gene expression analysis provides insights into molecular alterations associated with disease status. The incorporation of machine learning algorithms facilitates robust feature selection and prioritization of candidate biomarkers, offering the potential to enhance diagnostic accuracy beyond traditional approaches.

The research design encompasses a comprehensive workflow: first, systematic literature retrieval and bibliometric mapping to delineate key trends and knowledge gaps; second, integration of transcriptomic data from multiple publicly available databases to identify differentially expressed N-glycosylation-related genes in AD; third, employment of advanced machine learning techniques—including regularized regression and ensemble methods—to screen and validate potential biomarkers; and finally, network and regulatory analyses to elucidate interactions among identified genes, transcription factors (TFs), and molecular pathways. This multi-layered strategy aims to maximize the discovery of clinically relevant targets and provide a holistic understanding of the N-glycosylation landscape in AD.

## 2 Materials and methods

### 2.1 Data sources and retrieval strategy for N-glycosylation and AD bibliometrics

To investigate the intersection of N-glycosylation and AD through bibliometric analysis, data were retrieved from the Web of Science Core Collection, a gold-standard database for such research. English-language research articles and reviews published between 2001 and 2025 were included using the optimized search query:

“TS = ((“N-glycosylat*” OR “N-linked glycosylat*” OR “N-glycan*” OR “N-glycoprotein*” OR “protein N-glycosylat*” OR “n-glycoform*”) AND (Alzheimer* OR “Alzheimer’s disease” OR “Alzheimer’s” OR “Alzheimer disease” OR “Alzheimer’s-like” OR “Alzheimer pathology” OR “Alzheimer pathogenesis” OR “tau protein” OR “beta-amyloid”)).”

The initial search yielded 239 documents, which were filtered to retain only research articles and reviews. The complete list of these documents and detailed search outcomes are provided in Supplementary Table 1.

### 2.2 Analytical methods and tools for bibliometric analysis

Three analytical tools were employed: VOSviewer (v1.6.20), CiteSpace (v6.4 R1), and R (v4.3.3). VOSviewer was used to generate network visualizations, including thematic evolution mappings (to track shifts in research focus over time) and thematic quadrant mappings (to classify themes by centrality and density) (Zhou et al., 2025). CiteSpace constructed circular thematic landscapes (to identify clusters using silhouette coefficients) and performed targeted cluster analyses (for in-depth exploration of specific clusters) (Sebastian et al., 2020). Additionally, keyword burst detection was applied to characterize dynamic changes in research themes. R was utilized for quantitative analysis of publication data, including thematic trend quantification and keyword correlation calculations, to ensure rigorous examination of the literature.

### 2.3 Identification of N-glycosylation-related genes in AD via differential expression and machine learning

The GSE5281 dataset was analyzed to investigate N-glycosylation-related genes in AD. This dataset comprises gene expression profiles from 150 brain samples collected across three Alzheimer’s Disease Centers (ADCs): Arizona ADC, Duke University ADC, and Washington University ADC (Zhang et al., 2022). The dataset covers six brain regions relevant to AD and aging, including the entorhinal cortex, hippocampus, medial temporal gyrus, posterior cingulate, superior frontal gyrus, and primary visual cortex. Samples were stratified by diagnostic group, age group, and APOE genotype to ensure comprehensive coverage of molecular mechanisms in AD and normal aging.

Differentially expressed genes (DEGs) were identified using the LIMMA package in R, a widely used tool for microarray data analysis. The workflow included data preprocessing to normalize expression values and reduce technical variability, followed by fitting linear models to assess differential expression between AD-affected and control samples. DEGs were defined using stringent criteria: an absolute log2 fold change (logFc) > 0.4 and an adjusted *p*-value (Benjamini-Hochberg correction) < 0.05.

To investigate the overlap between DEGs and N-glycosylation-related genes, a list of N-glycosylation-associated genes was retrieved from the GeneCards database (Supplementary Table 1). The intersection between DEGs and this list was computed to identify genes implicated in both AD pathogenesis and N-glycosylation processes. All analyses, including data preprocessing, statistical modeling, and visualization, were performed using R.

Machine learning techniques were employed to identify genes associated with AD from an initial set of 39 genes derived from the intersection of DEGs and N-glycosylation-related genes in the GSE5281 and GSE122063 datasets. Three regularization methods were applied: Lasso, Elastic-Net, and Adaptive Lasso. Lasso regression was configured with an alpha parameter (α) set to 1, Elastic-Net with α = 0.5, and Adaptive Lasso without a predefined α, allowing adaptive adjustment of regularization strength. These methods were implemented using the glmnet package in R, with cross-validation to determine the optimal regularization parameter (λ) that minimized deviance. Genes with non-zero coefficients at the optimal λ were considered significant. Results were visualized using coefficient path plots and feature impact bar charts, and a Venn diagram was constructed to illustrate the overlap of selected genes across the three methods.

### 2.4 Identification of diagnostic biomarkers in DEGs via machine learning analysis

The relationship between five specific genes and AD was investigated using the GSE48350 and GSE5281 datasets. The workflow began with data preprocessing, including data normalization and removal of features with zero variance. Feature selection was performed using the Random Forest algorithm, configured to grow 100 trees to assess feature importance. Three machine learning models were constructed: logistic regression, Random Forest, and XGBoost. The logistic regression model was implemented using the “lrm” function from the “rms” package, the Random Forest model with 100 trees and importance parameter set to TRUE, and the XGBoost model trained for 50 rounds using the “xgb.train” function with the objective parameter set to “binary:logistic.” Model performance was evaluated using the Area Under the Curve (AUC) metric.

### 2.5 Identification of N-glycosylation-related genes associated with TMEM59 in AD

The GSE5281 and GSE122063 datasets were utilized to investigate molecular interactions associated with TMEM59, focusing on 11 genes implicated in N-glycosylation and AD. These genes—STT3A, MLEC, CALR, CANX, CTSD, MGAT1, MGAT2, MGAT3, MGAT5, APP, and STT3B—were selected based on their roles in glycosylation processes, calcium signaling, and protein quality control. For example, STT3A and STT3B are involved in glycosylation initiation (Lu et al., 2018), MGAT family members in glycan chain modification (Cha et al., 2017), and MLEC in glycan recognition (Dos Santos Silva et al., 2019). CALR and CANX act as chaperones in protein folding (Knee et al., 2003), while CTSD is linked to protein degradation (Marques et al., 2020). Eight feature selection algorithms were employed: LASSO, Elastic Net, Random Forest, XGBoost, Boruta, Stepwise Regression, Genetic Algorithm, and Decision Tree. Data preprocessing included normalization and removal of samples with missing values. The analysis was implemented in R with parallel computing for efficiency. Core genes selected by at least four algorithms were aggregated as the final output.

### 2.6 Discovering transcription factors linked to MLEC, TMEM59, and glial activation in AD

To identify pivotal TFs associated with AD, the ChIPBase database was leveraged to predict 44 TFs regulating the expression of four genes of interest: GFAP, FOS, MLEC, and TMEM59. These genes were selected based on their relevance to glial cell differentiation, a process intricately linked to AD pathogenesis (Giesen et al., 2003). The VennDiagram package in R was used to perform intersection analysis, identifying common TFs among GFAP, FOS, MLEC, and TMEM59.

Subsequently, the GSE5281 and GSE48350 datasets were analyzed, providing comprehensive gene expression profiles from brain regions relevant to AD and aging. Seven machine learning algorithms—Lasso, Elastic Net, Adaptive Lasso, XGBoost, Boruta, Genetic Algorithm, and Linear Discriminant Analysis (LDA)—were applied to assess the significance of the predicted TFs. Each algorithm was optimized for feature selection, balancing model complexity and predictive accuracy. The performance of these algorithms was quantified by the selection rate, defined as the ratio of selected genes to the total number of genes considered. Core biomarkers consistently recognized by at least four algorithms were identified as key contributors to AD pathogenesis. The R programming language was used for these analyses, with specific packages such as “glmnet,” “xgboost,” “Boruta,” “GA,” and “MASS” playing critical roles.

### 2.7 Investigating the diagnostic value of transcription factors associated with MLEC, TMEM59, and microglial activation in AD

The diagnostic performance of machine learning algorithms in AD was evaluated using a panel of genes associated with N-glycosylation and AD pathogenesis. The analysis focused on 13 molecular features: APP, CALR, CANX, CTSD, MAX, MGAT1, MGAT2, MGAT3, MGAT5, MLEC, STT3A, STT3B, and TMEM59. Machine learning algorithms, including logistic regression, support vector machines (linear and radial kernels), random forest, XGBoost, k-nearest neighbors (KNN), LDA, naive Bayes, and decision trees, were implemented to assess diagnostic accuracy. Feature selection was performed using random forest-based importance scoring, and models were trained and validated using five-fold repeated cross-validation. Key performance metrics, including AUC, accuracy, sensitivity, specificity, and F1 score, were calculated for each algorithm and gene combination. The analysis was conducted in R, leveraging packages such as “caret,” “pROC,” “xgboost,” and “randomForest.”

### 2.8 Machine learning and SHAP analysis of N-glycosylation-related molecular features in AD

The GSE5281 and GSE48350 datasets were used to analyze 13 molecular features selected based on their roles in N-glycosylation pathways and potential involvement in AD pathogenesis. These features included APP, CALR, CANX, CTSD, MAX, MGAT1, MGAT2, MGAT3, MGAT5, MLEC, STT3A, STT3B, and TMEM59. Data preprocessing involved normalization, median imputation for missing values, and removal of zero-variance features. Feature selection was conducted using a hybrid approach combining random forest importance scoring (ntree = 100) and logistic regression coefficients. Predictive models were built using random forest (ntree = 100), XGBoost (nrounds = 50), and logistic regression with interaction terms. Model performance was evaluated using AUC-ROC, with random forest and XGBoost achieving perfect AUC values of 1.0. SHAP values were calculated to interpret feature contributions, and molecular interactions were analyzed using heatmaps and interaction plots. All analyses were implemented in R, with visualization supported by packages such as “ggplot2,” “pheatmap,” and SHAP tools.

**FIGURE 1:**
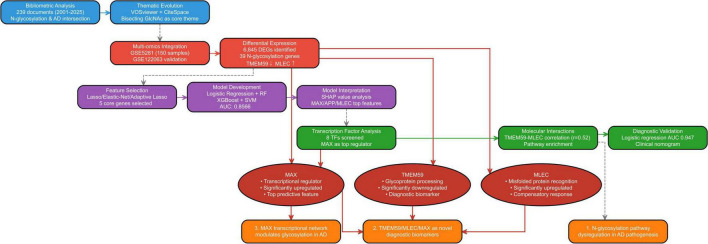
Workflow of the integrated bibliometric and bioinformatics analysis for identifying N-glycosylation-related genes in Alzheimer’s disease (AD).

The workflow implemented in our study is depicted in Figure 1, detailed accession numbers and information of the GEO datasets we used can be found in Supplementary Figure 1.

## 3 Results

### 3.1 Thematic evolution and research trends

**FIGURE 2:**
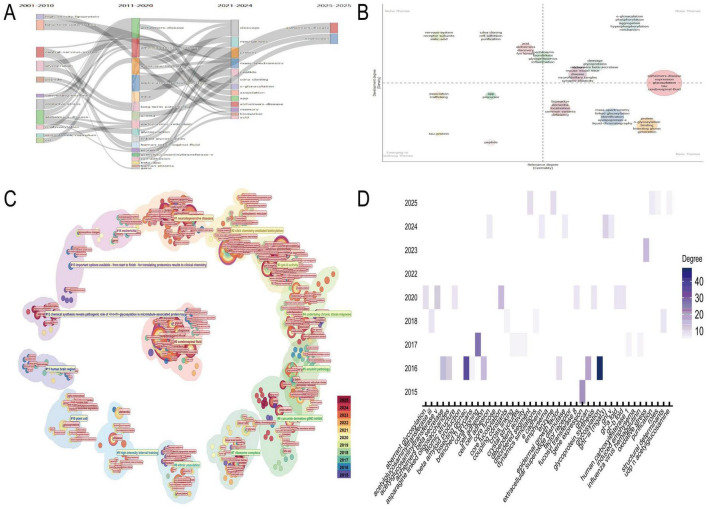
Thematic evolution and research trends. **(A)** The temporal progression of research themes in N-glycosylation and Alzheimer’s disease (AD) from 2001 to 2025, underscoring N-glycosylation as a pivotal theme in AD research. **(B)** Categorizes research priorities from 2021 to 2024 into four distinct categories: motor themes (high centrality and density), niche themes (specialized subfields), basic themes (foundational concepts), and emerging/declining themes. **(C)** Identifies key research clusters, including glycomic analysis of AD clinical samples and the role of GNT-III in glycan maturation. **(D)** Focuses on Cluster #3, highlighting keywords such as “glycosylation,” “glycan,” and “Alzheimer’s beta secretase,” and emphasizing the relevance of GNT-III to AD pathology.

Thematic evolution analysis of the N-glycosylation–AD field from 2001 to 2025 revealed sustained focus on AD’s interactions with core biological and biochemical processes, with N-glycosylation serving as a central connecting theme (Figure 2A). Between 2021 and 2024, research narrowed its focus to granular molecular and biochemical topics, including “cleavage,” “protein,” “mechanisms,” “mass-spectrometry,” and “peptide,” reflecting a shift toward dissecting AD pathogenesis at the molecular level to advance diagnostics and therapeutics. Thematic quadrant analysis (2021–2024) further clarified research priorities (Figure 2B): the upper-right quadrant (“Motor Themes”) included high-centrality and high-density topics such as “Alzheimer’s disease expression” and “glycosylation,” which bridge research directions; the upper-left quadrant (“Niche Themes”) featured specialized subfields like “nervous-system” and “receptor subunits”; the lower-right quadrant (“Basic Themes”) highlighted foundational concepts such as “biomarker” and “dementia”; and the lower-left quadrant (“Emerging/Declining Themes”) included evolving interests like “tau-protein” and “peptide.”

CiteSpace-generated circular thematic landscapes identified high-confidence clusters (Figure 2C): Cluster #0 (“cerebrospinal fluid,” “N-glycan profile,” silhouette coefficient: 0.726) emphasized glycomic analysis of AD clinical samples; Cluster #1 (“neurodegenerative diseases,” silhouette coefficient: 0.712) contextualized N-glycosylation within broader neurodegeneration; and Cluster #3 (“GNT-III activity,” silhouette coefficient: 0.909) focused on enzymatic regulation of glycan maturation. Nodes such as “cerebrospinal fluid” and “N-glycosylation” exhibited high centrality, underscoring their role in connecting clusters. Deep analysis of Cluster #3 revealed high-degree keywords—“glycosylation,” “glycan,” and “Alzheimer’s beta secretase”—highlighting their relevance to GNT-III–mediated AD pathways (Figure 2D). Temporal tracking (2015–2025) showed sustained interest in keywords such as “oxidative stress” and “cell adhesion,” indicating growing focus on GNT-III’s role in AD-related pathology.

### 3.2 Keyword analysis and research focus

**FIGURE 3:**
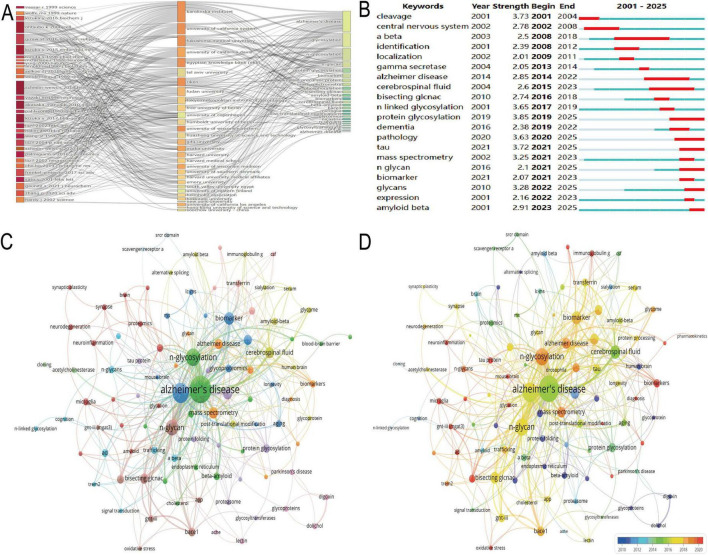
Keyword analysis and research focus. **(A)** Presents a three-field plot linking key studies, institutions, and keywords such as “glycosylation,” “Alzheimer’s disease,” and “amyloid-beta.” **(B)** Tracks keyword bursts, revealing early interest in “N-linked glycosylation” and recent focus on “pathology” and “tau.” **(C)** Displays a network visualization with “Alzheimer’s disease” as the central node. **(D)** Highlights recent thematic priorities via warm red node coloring, emphasizing “bisecting GlcNAc” and “GNT-III (MGAT3)” as emerging research focuses.

The three-field plot visualized the interconnections between key literature, institutions, and keywords in the N-glycosylation–AD field (Figure 3A). Influential studies, including “Vassar R (1999) Science,” “Kizuka Y (2016) Biochem J,” “Ohtsubo K (2006) Cell,” “Gizaw ST (2016) BBA-Gen Subjects,” and “Kizuka Y (2015) EMBO Mol Med,” formed the backbone of current understanding of N-glycosylation’s role in AD pathogenesis. These works were closely linked to leading institutions such as Karolinska Institutet, the University of California System, and Fukushima Medical University, underscoring their dominant contributions to the field. Core keywords included “glycosylation,” “Alzheimer’s disease,” and “amyloid-beta,” while “bisecting GlcNAc” emerged as a prominent focus, signaling growing interest in this specific glycan modification’s role in AD.

The keyword burst detection chart (Figure 3B) tracked high-impact temporal trends (2001–2025): early bursts included “N-linked glycosylation” (strength = 3.65) and “protein glycosylation” (strength = 3.85), reflecting foundational interest in glycan biology. More recently, “pathology” (strength = 3.63) and “tau” (strength = 3.72) have sustained bursts since 2020 (projected through 2025), indicating current focus on disease mechanisms. “Bisecting GlcNAc” also showed a notable burst (strength = 2.74) from 2016 to 2018, underscoring its historical relevance as a specialized research topic. Network visualizations further delineated thematic connections in N-glycosylation–AD research (Figures 3C,D). In Figure 3C, “Alzheimer’s disease” was the most central node (57 occurrences, total link strength = 134), reflecting its status as the field’s core focus, while “N-glycosylation” (24 occurrences, total link strength = 58) confirmed its pivotal role in AD pathological processes. Figure 3D highlighted recent thematic priorities via warm red node coloring: “N-glycosylation” (24 occurrences, link strength = 58) remained central, “Bisecting GlcNAc” (8 occurrences, link strength = 27) showed sustained relevance in recent studies, and “GNT-III (MGAT3)” (2 occurrences, link strength = 7) emerged as a novel focus, consistent with its role in regulating bisecting GlcNAc formation.

### 3.3 Key differentially expressed and N-glycosylation-related genes in Alzheimer’s disease identified via machine learning

**FIGURE 4:**
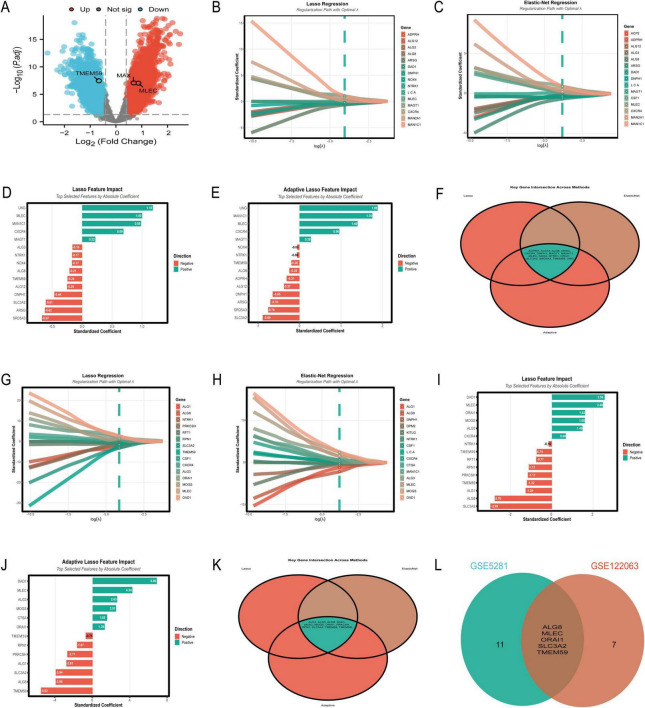
Key differentially expressed and N-glycosylation-related genes. **(A–F)** Focus on the GSE5281 dataset: a volcano plot **(A)** highlights significant upregulated (red) and downregulated (blue) differentially expressed genes (DEGs); regression coefficient paths and feature impact bar charts from Lasso **(B,D)**, Elastic-Net **(C)**, and Adaptive Lasso **(E)** reveal genes such as UNG, MLEC, and MAN1C1 as top contributors; a Venn diagram **(F)** underscores overlapping genes across these methods, identifying robust biomarker candidates. **(G–L)** Shift to the GSE122063 dataset: regression coefficient paths and feature impact bar charts **(G–J)** highlight DAD1, MLEC, and TMEM59 as key genes; a Venn diagram **(K)** illustrates overlapping genes across methods; comparative analysis **(L)** reveals consistent candidates such as MLEC and TMEM59 between the two datasets.

Differential expression analysis of the GSE5281 dataset identified 6,845 differentially expressed genes (DEGs) (Supplementary Table 1), visualized in the volcano plot (Figure 4A). The plot highlights significant upregulated genes (red) and downregulated genes (blue), with gray dots representing non-significant genes. Notably, TMEM59 was identified as a significantly downregulated gene, while MLEC and MAX were significantly upregulated in AD-affected samples compared to controls. The intersection of DEGs with N-glycosylation-related genes from the GeneCards database yielded 39 overlapping genes.

In Figure 4B, the Lasso regression coefficient path reveals the influence of various genes on the model, with genes like UNG and MLEC showing the most significant impact as indicated by their steep paths. Figure 4C presents the Elastic-Net regression coefficient path, where genes such as UNG, MLEC, and MAN1C1 are prominently featured, demonstrating their importance in the model with considerable standardized coefficients. Figure 4D showcases the Lasso feature impact, highlighting the top genes selected by absolute coefficient. UNG, MLEC, and MAN1C1 are at the forefront, with UNG having the highest positive coefficient, suggesting its strong association with the disease. Figure 4E illustrates the Adaptive Lasso feature impact, where UNG again leads with the highest positive coefficient, followed by MAN1C1 and MLEC. This figure emphasizes the genes’ significance in the context of AD, with a clear distinction between positive and negative contributions. Lastly, Figure 4F provides a Venn diagram of the key gene intersections across the three methods, showing a core set of genes that are consistently selected: ADPRH, ALG12, ALG8, ARSG, CXCR4, DNPH1, MAGT1, MAN1C1, MLEC, NOX4, NTRK1, ORAI1, SLC3A2, SRD5A3, TMEM59, and UNG. This overlap underscores the robustness of these genes as potential biomarkers for AD, as they are identified across different regularization techniques.

The analysis of the GSE122063 dataset using machine learning algorithms identified a set of genes with potential significance in AD. Figure 4G illustrates the Lasso regression results, where genes such as DAD1 and MLEC exhibit the highest absolute standardized coefficients, indicating their strong influence on the model. Figure 4H presents the Elastic-Net regression outcomes, showing a similar pattern with DAD1 and MLEC being prominent, alongside other genes like ALG8 and MOGS. Figure 4I highlights the top features selected by Lasso regression, with DAD1, MLEC, and ALG8 having the most substantial impact. The Adaptive Lasso results in Figure 4J show DAD1 as the most influential gene, followed by TMEM59 and MLEC, pointing to their crucial role in the disease pathology. The Venn diagram in Figure 4K reveals the intersection of genes selected by all three methods, with ALG1, ALG3, ALG8, DAD1, MLEC, MOGS, ORAI1, PRKCSH, RPN1, SLC3A2, TMEM59, and TMEM59L being consistently identified across Lasso, Elastic-Net, and Adaptive Lasso, underscoring their potential as biomarkers. Finally, Figure 4L compares the gene selection between the GSE5281 and GSE122063 datasets, showing an overlap in ALG8, MLEC, ORAI1, SLC3A2, and TMEM59, which further validates their importance in AD research.

### 3.4 Validation of gene biomarkers in DEGs and nomogram development for Alzheimer’s disease diagnosis

**FIGURE 5:**
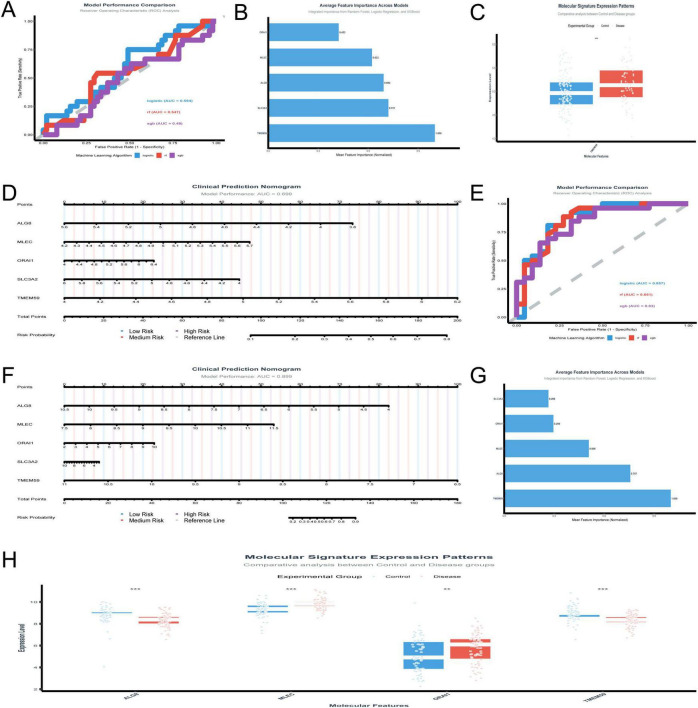
Validation of gene biomarkers and diagnostic models. **(A–D)** Analyze the GSE48350 dataset: model performance is compared across machine learning algorithms **(A)**; TMEM59 emerges as the most important gene through rankings **(B)**; molecular signature expression patterns distinguish control and disease groups **(C)**; a clinical prediction nomogram **(D)** validates TMEM59’s role. **(E–H)** Focus on the GSE5281 dataset: logistic regression and Random Forest models achieve AUC scores of 0.8566 and 0.8505 **(E)**; a nomogram **(F)** achieves an AUC of 0.899, emphasizing TMEM59’s contribution; feature importance analysis **(G)** and expression patterns **(H)** highlight TMEM59 and MLEC as critical drivers. *p < 0.05, **p < 0.01, ***p < 0.001.

The analysis of the GSE48350 dataset focusing on five genes yielded several significant findings. The logistic regression model achieved a test AUC of 0.594, indicating moderate predictive accuracy. Figure 5A presents the model performance comparison across various machine learning algorithms, showcasing the logistic regression model’s moderate predictive power. Figure 5B illustrates the ranking of the five genes by their importance across different models, with TMEM59 showing the highest mean importance. Figure 5C depicts the molecular signature expression patterns, highlighting a clear distinction between control and disease groups, particularly for TMEM59, which exhibited the most pronounced differential expression. Figure 5D presents the clinical prediction nomogram, which achieved a model performance AUC of 0.699, indicating a reasonable ability to discriminate between control and disease states. This nomogram assigns points to each gene based on its standardized coefficient, with TMEM59 receiving the highest point allocation, significantly contributing to the total points and thus the risk probability of AD. These results underscore the potential utility of these genes as diagnostic biomarkers for AD, with TMEM59 emerging as a particularly promising candidate due to its significant differential expression and high feature importance across models.

The in-depth analysis of the GSE5281 dataset shed light on the roles of five key genes—ALG8, MLEC, ORAI1, SLC3A2, and TMEM59—in AD. The performance of various machine learning models was evaluated in Figure 5E, with logistic regression and Random Forest standing out, achieving test AUC scores of 0.8566 and 0.8505, respectively. XGBoost also performed well, with a test AUC of 0.8304. The clinical prediction nomogram in Figure 5F, which incorporates these genes, shows a high model performance AUC of 0.899, indicating its strong predictive power. This nomogram assigns points to each gene based on its expression level, with TMEM59 receiving the most points, suggesting it plays a crucial role in determining the risk probability of AD. Figure 5G highlights the average feature importance across models, with TMEM59 being identified as the most important gene, followed by ALG8 and MLEC. This underscores the significant impact of these genes on the model’s predictive capabilities. Figure 5H, which includes MLEC and TMEM59, shows molecular signature expression patterns.

### 3.5 Characterization of N-glycosylation-related genes correlated with TMEM59 in Alzheimer’s disease pathology

**FIGURE 6:**
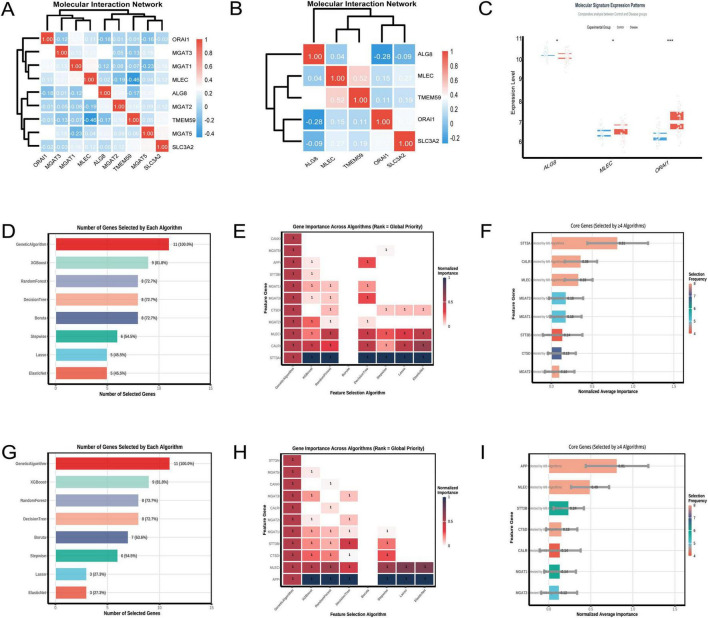
Molecular interactions of TMEM59. **(A)** (GSE5281) shows a heatmap revealing a strong negative correlation between TMEM59 and MLEC, with hierarchical clustering distinguishing their expression profiles. **(B)** (GSE122063) highlights a moderate positive correlation between these genes. Panel **(C)** displays elevated MLEC expression in disease groups within GSE122063. **(D–F)** (GSE122063) detail gene selection rates, noting the Genetic Algorithm’s 100% selection rate, and illustrate the importance of genes like STT3A, CALR, and MLEC across algorithms. **(G–I)** (GSE5281) reveal similar trends, with the Genetic Algorithm again achieving a 100% selection rate and underscoring genes like APP, MLEC, and STT3B. Overall, MLEC consistently ranks as a key gene associated with TMEM59 across both datasets. *p < 0.05, **p < 0.01, ***p < 0.001.

The heatmap (Figure 6A) resulting from the analysis of the GSE5281 dataset illustrates the pairwise correlations among the nine selected genes. A significant negative correlation of –0.46 was observed between TMEM59 and MLEC, as indicated by the deep blue color in the heatmap. This finding suggests an inverse relationship between the expression levels of these two genes. The hierarchical clustering dendrogram positioned TMEM59 and MLEC in separate clusters, reinforcing the distinctness of their expression profiles among the analyzed genes. In the analysis of the GSE122063 dataset (Figure 6B), a notable correlation was identified between TMEM59 and MLEC, with a correlation coefficient of 0.52, indicating a moderate positive relationship. This finding suggests that the expression levels of TMEM59 and MLEC may be associated in the samples analyzed from individuals with AD. Analyzing the GSE122063 dataset, Figure 6C shows that MLEC expression levels are increased in the disease group compared to controls.

In the GSE122063 dataset, the feature selection algorithms identified STT3A, CALR, and MLEC as the top three genes associated with TMEM59. Figure 6D shows that the Genetic Algorithm selected all genes, achieving a 100% selection rate, while XGBoost selected 9 genes, indicating an 81.8% selection rate. The heatmap in Figure 6E demonstrates the normalized importance of each gene across algorithms, with STT3A, CALR, and MLEC showing the highest importance. Figure 6F summarizes the core genes, highlighting STT3A, CALR, and MLEC as frequently selected by multiple algorithms. In the GSE5281 dataset, the top three genes associated with TMEM59 were APP, MLEC, and STT3B. Figure 6G details the number of genes selected by each algorithm, with the Genetic Algorithm achieving a 100% selection rate and XGBoost selecting 9 genes, indicating an 81.8% selection rate. The heatmap in Figure 6H illustrates the normalized importance of each gene across algorithms, with APP, MLEC, and STT3B consistently showing high importance. Figure 6I confirms these findings, emphasizing APP, MLEC, and STT3B as core genes frequently selected by multiple algorithms. Across both datasets, MLEC is consistently identified as one of the top three genes associated with TMEM59.

### 3.6 Uncovering transcription factors that regulate MLEC, TMEM59, and glial activation in Alzheimer’s disease

**FIGURE 7:**
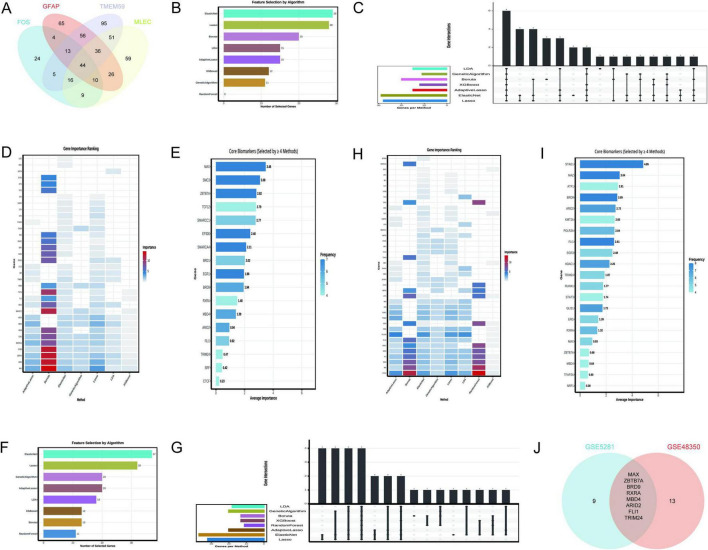
Transcription factors regulating N-glycosylation-related genes. **(A)** Identifies 44 shared transcription factors (TFs) among GFAP, FOS, MLEC, and TMEM59. **(B–E)** Focus on GSE5281: **(B)** Elastic Net and Lasso have the highest selection rates. **(C)** Compares selection rates, **(D)** Ranks TF importance via a heatmap, and **(E)** highlights core biomarkers like MAX. **(F–I)** Analyze GSE48350: **(F)** Indicates Elastic Net selected the most genes (37), **(G)** Contrasts selection rates, **(H)** ranks TF importance, and Panel I shows selection frequency. **(J)** Presents a Venn diagram of key TFs from both datasets, identifying eight common factors: MAX, ZBTB7A, BRD9, RXRA, MBD4, ARID2, FLI1, and TRIM24.

The Venn diagram analysis identified 44 shared TFs among the genes GFAP, FOS, MLEC, and TMEM59 (Figure 7A), as detailed in Supplementary Table 1. This set of TFs is indicative of a regulatory network that may be involved in the biological processes associated with these genes. The rigorous application of machine learning algorithms to the GSE5281 dataset has yielded a refined list of 17 key TFs from an initial pool of 44 predicted TFs. This selection was based on the consistent recognition by multiple algorithms, indicating their potential significance in AD. The 17 TFs identified as critical include MAX, SMC3, ZBTB7A, TCF12, SMARCC1, EP300, SMARCA4, BRD1, EGR1, BRD9, RXRA, MBD4, ARID2, FLI1, TRIM24, SRF, and CTCF. As depicted in Figure 7B, the Elastic Net and Lasso algorithms demonstrated the highest gene selection rates, suggesting their effectiveness in this context. Figure 7C provides a comparative view of the number of genes selected by each method, highlighting the variability in gene selection across different algorithms. Figure 7D presents a heatmap that ranks gene importance across various methods, offering a visual representation of the relative significance of each TF. Figure 7E specifically emphasizes the core biomarkers (MAX ranked top), selected by at least four methods, underscoring their potential role as key regulators in AD.

The analysis leveraging the GSE48350 dataset, as depicted in Figure 7F, demonstrates that the Elastic Net algorithm selected the highest number of genes (37), followed by Lasso (32), and Genetic Algorithm and Adaptive Lasso (20 each). In contrast, the Random Forest and XGBoost algorithms selected fewer genes, indicating a lower frequency of gene importance attribution by these methods. Figure 7G provides a comparative overview of the number of genes selected by each method, with the bar chart visually representing the variability in gene selection across different algorithms. Figure 7H presents a heatmap that ranks gene importance across various methods, offering a visual representation of the relative significance of each TF. Notably, genes such as STAG1, MAZ, BRD9, and FLI1 were consistently identified as important across multiple methods, as highlighted in Figure 7I, which also shows the frequency of gene selection by the algorithms. The Venn diagram in Figure 7J illustrates the intersection of key TFs identified from two distinct datasets, GSE5281 and GSE48350. The intersection, indicating the common TFs significant to both datasets, consists of 8 factors: MAX, ZBTB7A, BRD9, RXRA, MBD4, ARID2, FLI1, and TRIM24.

### 3.7 Evaluating the diagnostic potential of transcription factors linked to MLEC, TMEM59, and microglial activation in Alzheimer’s disease

**FIGURE 8:**
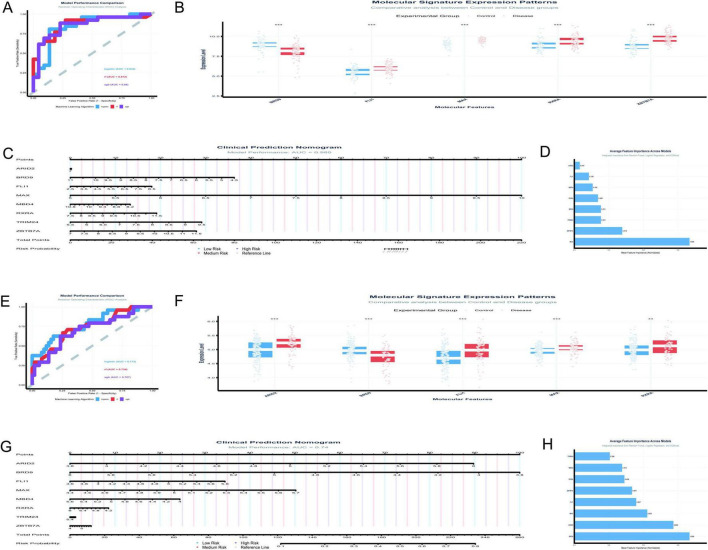
Diagnostic potential of transcription factors. **(A–D)** Analyze the GSE5281 dataset: ROC curves compare predictive models **(A)**, with Random Forest achieving the highest AUC of 0.8715; boxplots reveal significant differences in TF expression between control and disease groups **(B)**; a clinical prediction nomogram **(C)** achieves an AUC of 0.985; feature importance analysis **(D)** ranks MAX as the most influential TF. **(E–H)** Focus on the GSE48350 dataset: logistic regression demonstrates the highest AUC of 0.772 **(E)**; differential expression analysis supports these findings **(F)**; a nomogram achieves an AUC of 0.74 **(G)**; feature importance analysis highlights significance in BRD9 and MAX **(H)**. *p < 0.05, **p < 0.01, ***p < 0.001.

The analysis of the GSE5281 dataset (Figures 8A–D) has been conducted with a focus on eight TFs, utilizing machine learning models to predict Alzheimer’s disease risk. The results are summarized as follows: Figure 8A presents the ROC curves for three predictive models. The logistic regression model achieved a test AUC of 0.8444, indicating good model performance. The random forest model demonstrated a slightly higher discriminative ability with a test AUC of 0.8715, while the XGBoost model showed a test AUC of 0.8601. These AUC values suggest that all models are effective in distinguishing between disease and control samples, with the random forest model performing the best among the three. In Figure 8B, the boxplot analysis reveals significant differences in the expression levels of TFs between control and disease groups. MAX, in particular, shows a notable higher median expression in control samples compared to disease samples, suggesting its potential role in the disease’s pathogenesis.

Figure 8C features a clinical prediction nomogram that integrates the influence of various TFs to estimate disease risk. The nomogram is based on a logistic regression model with an AUC of 0.985, indicating excellent predictive power. For instance, a patient with a MAX expression level of 6.5 would receive 30 points, which, when combined with points from other factors, contributes to a total score that predicts disease risk. Figure 8D illustrates the average feature importance across the three models, with MAX being the most influential, followed by ZBTB7A, BRD9, and RXRA. The normalized mean importance scores highlight MAX’s predominant role in the model’s predictive accuracy, with a score of 1.000, significantly higher than the other factors.

The current study (Figures 8E–H) analyzed the expression patterns and diagnostic potential of eight TFs (MAX, ZBTB7A, BRD9, RXRA, MBD4, ARID2, FLI1, and TRIM24) in AD using the GSE48350 dataset, which comprises 253 samples from postmortem brain tissues. Figure 8E illustrates the receiver operating characteristic (ROC) curves for the three machine learning models. The logistic regression model demonstrated the highest area under the curve (AUC) of 0.772, followed by RF (AUC = 0.734) and XGBoost (AUC = 0.707). The performance gap between training and testing datasets was minimal for all models, indicating robust generalizability. Figure 8F presents the differential expression analysis of five TFs (ARID2, BRD9, FLI1, MAX, and RXRA) between control and disease groups. Significant differences in expression levels were observed for all analyzed TFs. Notably, MAX exhibited elevated expression in the disease group compared to the control group. Figure 8G displays a clinical prediction nomogram based on the logistic regression model, with an AUC of 0.74. The nomogram assigns points to each TF based on its expression level, and the total points are converted into a risk probability. MAX contributes significantly to the risk score, alongside BRD9 and ARID2. Specifically, higher expression levels of MAX are associated with an increased risk probability, underscoring its role as an important predictor in the model. Figure 8H highlights the average feature importance of the eight TFs across the three machine learning models. BRD9 ranked highest with a normalized importance of 1.000, followed by ARID2 (0.860) and MAX (0.633). The integrated importance scores reflect the relative contribution of each TF to the diagnostic model. MAX emerges as one of the most critical features, with its importance supported by both its differential expression and its substantial contribution to the model’s predictive power.

### 3.8 Optimizing diagnostic accuracy with key molecules (MAX, MLEC, TMEM59) and N-glycosylation-related genes across different algorithms

**FIGURE 9:**
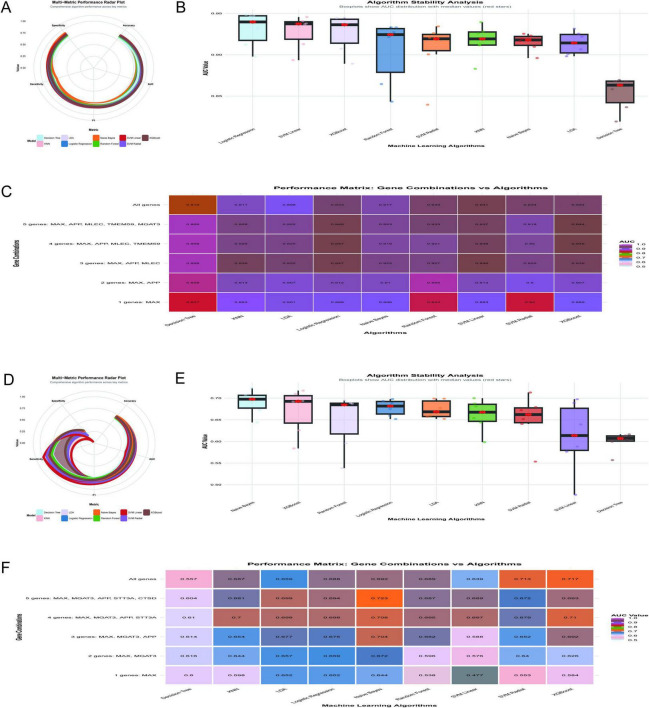
Optimization of diagnostic models. **(A–C)** Analyze the GSE5281 dataset: a radar plot compares key performance metrics across algorithms **(A)**, with logistic regression demonstrating superior accuracy; AUC stability is assessed through boxplots **(B)**, further validating logistic regression and SVM linear; a performance matrix heatmap **(C)** underscores logistic regression’s efficiency, achieving an AUC of 0.947 with three genes (MAX, APP, MLEC). **(D–F)** Shift to the GSE48350 dataset: Naive Bayes achieves the highest mean AUC of 0.723 using five genes **(D)**; algorithm stability is highlighted **(E)**; performance across gene combinations is shown in heatmap matrices **(F)**.

The diagnostic performance of machine learning algorithms was evaluated using the GSE5281 dataset, with a focus on achieving optimal diagnostic efficacy with fewer gene combinations. Figure 9A illustrates a radar plot comparing key performance metrics across algorithms. Logistic regression demonstrated superior performance in accuracy, specificity, and F1 score, while XGBoost and SVM linear showed strong AUC values. Figure 9B highlights algorithm stability through boxplots of AUC distributions. Logistic regression and SVM linear exhibited the highest median AUC values with minimal variability, indicating robustness. Figure 9C displays a performance matrix heatmap for different gene combinations and algorithms. Notably, logistic regression achieved an AUC of 0.947 using only three genes (MAX, APP, MLEC), and an AUC of 0.948 with five genes (MAX, APP, MLEC, TMEM59, MGAT3). Even with a single gene (MAX), logistic regression demonstrated an AUC of 0.898, underscoring its diagnostic utility. The findings highlight the importance of MAX as a critical feature in gene panels, with logistic regression emerging as the most stable and accurate algorithm for minimizing the number of genes while maximizing diagnostic efficacy.

The analysis of the GSE48350 dataset, comprising 253 samples, evaluated the diagnostic performance of machine learning algorithms across different gene combinations related to N-glycosylation and AD. Figure 9D illustrates the multi-metric performance of algorithms, with Naive Bayes achieving the highest mean AUC of 0.723 using the five-gene combination of MAX, MGAT3, APP, STT3A, and CTSD, supported by an accuracy of 0.702, sensitivity of 0.873, specificity of 0.331, and an F1 score of 0.8. Figure 9E shows the stability of algorithms through boxplots of AUC distributions, with Naive Bayes demonstrating robust stability with a median AUC above 0.70, while algorithms such as Decision Tree and SVM Linear exhibited greater variability. Figure 9F provides a heat map matrix comparing algorithm performance across gene combinations, indicating that Naive Bayes achieved the highest AUC of 0.723 with five genes (MAX, MGAT3, APP, STT3A, CTSD). Even with fewer genes, Naive Bayes maintained strong diagnostic efficacy, achieving an AUC of 0.704 with three genes (MAX, MGAT3, APP) and an AUC of 0.644 with a single gene (MAX). While the five-gene combination yielded the highest diagnostic performance, the three-gene combination offers a balance between diagnostic accuracy and clinical practicality. The MAX gene was identified as the most critical single gene in this analysis.

### 3.9 Key molecular features and interactions in Alzheimer’s disease revealed by machine learning and SHAP analysis

**FIGURE 10:**
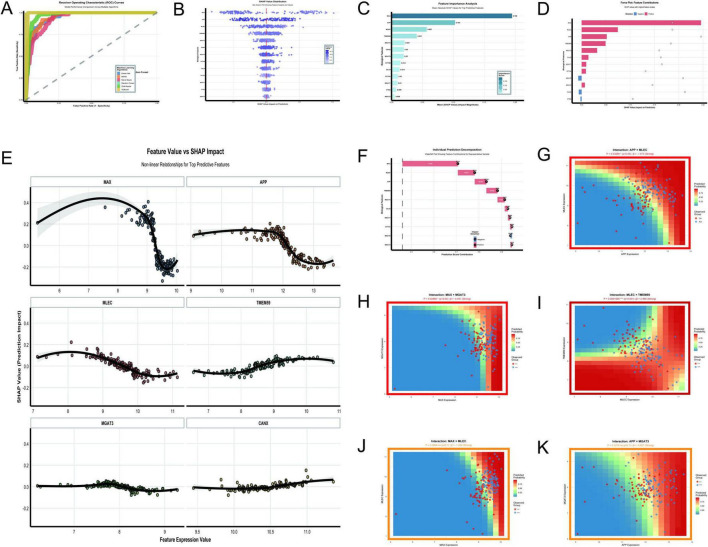
Key molecular features and interactions. **(A–K)** Focus on the GSE5281 dataset: ROC curves demonstrate Random Forest and XGBoost achieving perfect AUC values of 1.0 **(A)**; SHAP value distribution **(B)** and feature importance analysis **(C)** identify MAX, APP, MLEC, and TMEM59 as top predictors; a force plot **(D)** emphasizes their dominant roles in driving prediction scores. **(E–K)** Explore nonlinear relationships and significant interactions: interactions include a positive effect between MLEC and TMEM59 (*p* = 0.00019) and a negative effect between MAX and MGAT3 (*p* = 0.0288).

The analysis of Figures 10A–K provides a comprehensive overview of the key molecular features and interactions driving AD classification in the GSE5281 dataset. The random forest and XGBoost models demonstrated exceptional performance with perfect AUC values of 1.0, outperforming other algorithms such as SVM Radial (AUC = 0.976) and Elastic Net (AUC = 0.953) (Figure 10A). Feature importance analysis identified MAX, APP, MLEC, and TMEM59 as the most impactful predictors, with MAX showing the highest mean absolute SHAP value of 0.195 (Figures 10B,C). The SHAP value distribution (Figure 10B) and feature value vs. SHAP impact analysis (Figure 10E) revealed nonlinear relationships between feature expression levels and their contributions to predictions. MAX and APP exhibited strong positive impacts, with their contributions increasing sharply at higher expression levels. MLEC and TMEM59 also showed significant contributions, though their dependencies on expression levels were weaker. The force plot (Figure 10D) and individual prediction decomposition (Figure 10F) further highlighted the dominant roles of MAX, MLEC, APP, and TMEM59 in driving prediction scores, with MAX contributing the most significantly. Interaction analysis uncovered several key relationships. The interaction between MLEC and TMEM59 was highly significant (*p* = 0.00019, β = 2.469), with higher expression of both genes correlating with increased predicted probabilities (Figure 10I). Conversely, the interaction between MAX and MGAT3 (*p* = 0.0288, β = –4.491) showed a strong negative effect, where higher MAX expression reduced predicted probabilities when MGAT3 expression was low (Figure 10H). The interaction between APP and MLEC (*p* = 0.03068, β = –1.473) also demonstrated a significant negative effect (Figure 10G). While some interactions, such as MAX × MLEC (*p* = 0.3604, β = –1.439) and APP × MGAT3 (*p* = 0.3216, β = –0.827), did not reach statistical significance, they suggested potential regulatory trends worth further investigation (Figures 10J,K).

**FIGURE 11:**
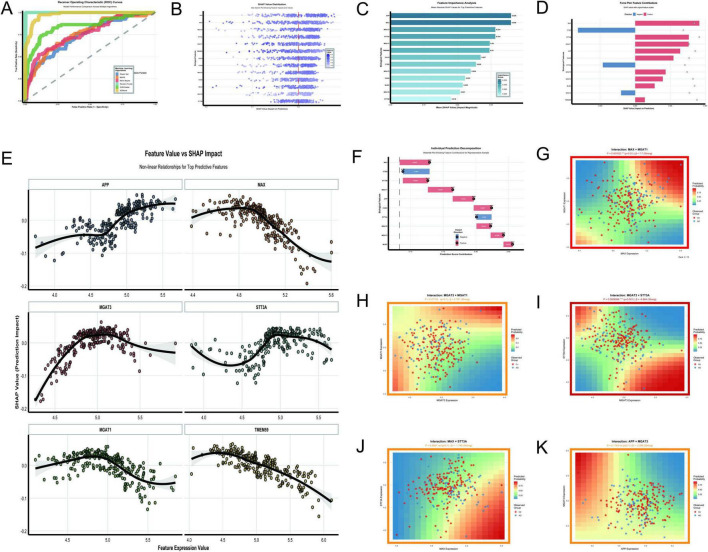
N-glycosylation-related molecular features. **(A–K)** Analyze the GSE48350 dataset: ROC curves highlight Random Forest’s perfect AUC of 1.0 **(A)**; SHAP value distribution **(B)** and feature importance analysis **(C)** underscore APP and MAX as top predictors. **(D–F)** Detail feature contributions: a force plot **(D)** and waterfall plot **(F)** detail feature contributions to prediction scores. **(G–K)** Explore significant interactions: relationships include a positive effect between MAX and MGAT1 (*p* = 0.0016) and a negative effect between MGAT3 and STT3A (*p* < 0.001).

The analysis of N-glycosylation-related molecular features in AD using the GSE48350 dataset provided a comprehensive understanding of their roles and interactions through 11 key visualizations. The Random Forest model demonstrated exceptional classification performance with an AUC of 1.0 (Figure 11A), outperforming other algorithms such as XGBoost (AUC = 0.976) and SVM Radial (AUC = 0.852). This result was supported by SHAP value distributions (Figure 11B), which identified APP and MAX as the most impactful features due to their high SHAP values. The feature importance analysis (Figure 11C) further confirmed APP and MAX as the top predictors, followed by MGAT3 and STT3A. Force plots (Figure 11D) and waterfall plots (Figure 11F) detailed the directional contributions of these features to prediction scores, with MAX showing a strong positive impact and CTSD a significant negative impact. The non-linear relationships between feature expression values and SHAP impacts (Figure 11E) revealed that APP and MAX had positive correlations with AD classification, while STT3A demonstrated a negative correlation. MGAT3 exhibited a non-linear trend, highlighting its complex role in AD pathology. Interaction analyses uncovered significant synergies between molecular pairs. The interaction between MAX and MGAT1 (Figure 11G) showed a strong positive effect (*P* = 0.0016, β = 7.7), while the interaction between MGAT3 and STT3A (Figure 11I) demonstrated a highly significant negative effect (*P* < 0.001, β = –6.864). The interaction between MGAT3 and MGAT1 (Figure 11H) also showed a near-significant effect (*P* = 0.0775, β = 2.767). Other interactions, such as APP × MGAT3 (Figure 11K) and MAX × STT3A (Figure 11J), showed limited or non-significant effects (*P* = 0.1743, β = –2.298 and *P* = 0.4841, β = –1.745, respectively).

## 4 Discussion

AD is a devastating neurodegenerative disorder characterized by progressive cognitive decline and memory impairment, predominantly affecting the elderly population (Rampa et al., 2013). Its pathophysiology is marked by the accumulation of amyloid-beta plaques and neurofibrillary tangles, which lead to synaptic dysfunction and neuronal loss (Han and Shi, 2016). As the leading cause of dementia globally, AD poses significant challenges to affected individuals, their families, and healthcare systems (Lozupone et al., 2025). With its prevalence on the rise, understanding the underlying mechanisms of AD and identifying reliable biomarkers for early diagnosis have become critical priorities in the development of effective therapeutic strategies (Wu et al., 2021).

This investigation explores the role of N-glycosylation in AD pathology, highlighting its potential as a key factor in disease progression. By integrating gene expression data from the multiple datasets with advanced machine learning techniques, we identified several N-glycosylation-associated genes that may serve as promising biomarkers for early detection. These findings underscore the importance of these biomarkers in improving diagnostic accuracy and guiding future therapeutic interventions, thereby advancing our understanding of AD mechanisms and paving the way for targeted treatment approaches.

In-depth analysis of gene expression profiles in the GSE5281 dataset revealed a substantial set of DEGs. Notably, TMEM59 was markedly downregulated, while MLEC and MAX were significantly upregulated. This observation aligns with transcriptomic studies indicating widespread alterations in glycosylation-related genes in the brains of individuals with AD, where nearly 80% of such genes were reported to be differentially expressed in at least one brain region (Tang et al., 2023). The downregulation of TMEM59 may disrupt glycoprotein maturation and trafficking, impairing protein-folding quality control mechanisms and exacerbating proteostatic stress—a phenomenon previously linked to AD pathology (Meng et al., 2020). Conversely, the upregulation of MLEC, which encodes malectin, an ER-resident lectin involved in the recognition of misfolded glycoproteins, could reflect a compensatory response to the increased burden of unfolded proteins or aberrant N-glycan structures in the AD brain (Chen et al., 2011). MAX, a transcriptional regulator implicated in cellular responses to metabolic stress, may be associated with the metabolic alterations observed in AD neurons (Hsieh and Dang, 2016). These expression patterns diverge from those seen in other neurodegenerative conditions, underscoring a potential disease-specific adaptation in N-glycosylation pathways. Thus, the coordinated dysregulation of TMEM59, MLEC, and MAX highlights a mechanistic nexus linking glycoprotein homeostasis, ER stress, and metabolic reprogramming in AD.

The application of machine learning algorithms enabled the identification of 5 N-glycosylation-associated genes with potential diagnostic relevance for AD. Feature selection methods such as Lasso, Elastic-Net, and Adaptive Lasso were employed to address concerns raised in previous literature regarding the limitations of single-method feature selection, particularly in high-dimensional, collinear “omics” data. Recent biomarker studies have emphasized the importance of integrating multiple selection strategies to enhance the robustness and interpretability of candidate biomarkers, especially when dataset shift and cohort heterogeneity pose significant translational challenges. Unlike the reliance on univariate or standard regression-based selections, the present methodology combines penalized regression with ensemble learning, thereby mitigating overfitting and improving out-of-sample predictive performance. This integrative analytic framework surpasses previous approaches by systematically accounting for correlation structures among glycosylation genes and accommodating the biological complexity inherent to AD. Importantly, the genes prioritized by this approach show minimal overlap with those identified by conventional differential expression analysis alone, demonstrating the capacity of machine learning to uncover non-obvious biomarker candidates in the context of multifactorial disease etiology (Abbas and El-Manzalawy, 2020).

The observed correlation between MLEC and TMEM59 suggests a functional interplay within the N-glycosylation pathway, which may be pivotal for maintaining glycoprotein quality control in neural tissue. Previous glycomic investigations have identified enhanced or aberrant glycan biosynthesis as a hallmark of AD, wherein genetic or dietary modulation of glycosylation enzymes directly impacts cognitive and behavioral phenotypes (Hawkinson et al., 2025). The concurrent upregulation of MLEC alongside the downregulation of TMEM59 could indicate an adaptive but ultimately insufficient cellular response to glycoprotein misfolding and aggregation. Mechanistically, MLEC may facilitate the recognition and retention of misfolded glycoproteins in the endoplasmic reticulum, whereas TMEM59 is implicated in the trafficking and processing of these substrates (Boada-Romero et al., 2013). The linkage between MLEC and TMEM59 thus provides a molecular rationale for the dysfunction in protein quality control and aberrant glycosylation characterizing AD neuropathology.

The identification of eight TFs—including MAX, ZBTB7A, BRD9, RXRA, MBD4, ARID2, FLI1, and TRIM24—as regulators of MLEC, TMEM59, GFAP, and FOS expands our understanding of the gene regulatory networks that orchestrate glycosylation and neuroinflammatory responses in AD. Prior computational studies have highlighted the centrality of specific TFs, such as STAT1 and HSF5, in controlling glycosyltransferase gene expression in the AD brain (Tang et al., 2023), and these regulatory axes are predicted to alter glycan biosynthesis in a cell-type and region-specific manner. The selection of MAX as a pivotal regulator is particularly noteworthy, as its role extends beyond classic metabolic regulation, implicating it in neural stress responses and possibly in the modulation of microglial and astrocytic activation states (McFerrin and Atchley, 2011). Notably, this regulatory constellation diverges from previously established transcriptional landscapes in other neurodegenerative disorders, suggesting a unique transcriptional architecture underpinning AD-specific glycosylation and glial responses. These findings not only corroborate the significance of transcriptional modulation in AD pathogenesis but also reveal novel candidate regulators that may serve as intervention points for modulating disease trajectory.

The comparative evaluation of diagnostic algorithms demonstrated that logistic regression exhibited superior accuracy and stability for classifying AD status based on gene expression signatures, outperforming ensemble methods such as random forest and XGBoost in this context. This result stands in partial contrast to recent meta-analyses of machine learning in medical diagnostics, which often report that ensemble models provide enhanced performance, especially when handling high-dimensional or nonlinear data. However, the relatively modest sample sizes and stringent feature selection employed in this study may favor the generalizability and interpretability of linear models, aligning with reports that logistic regression can outperform more complex algorithms when the number of predictors is tightly controlled and the risk of overfitting is minimized (Hu and Hu, 2014). Furthermore, the consistent performance of logistic regression across external validation datasets underscores its practical utility in clinical biomarker development for AD, particularly when transparency and reproducibility are paramount. This methodological insight supports a nuanced approach to model selection, emphasizing context-dependent optimization over blanket preference for algorithmic complexity.

The limitations of this study are primarily reflected in the absence of wet lab validation and the relatively small sample size, which restricts the generalizability of the findings. The reliance on publicly available datasets may introduce batch effects that could skew the results, potentially impacting the robustness of the identified biomarkers (Carry et al., 2023). Additionally, while machine learning techniques were employed to enhance the identification of N-glycosylation-related genes, the interpretability of complex models remains a challenge, necessitating further validation to establish clinical relevance. These aspects highlight the need for larger, multi-center studies that integrate both computational and experimental approaches to bolster the reliability of the findings.

In summary, this investigation identified several genes associated with N-glycosylation and AD, demonstrating their potential as biomarkers for early diagnosis. The innovative application of machine learning methods underscores the significance of these findings in advancing our understanding of AD pathology and facilitating early intervention strategies. Future research should prioritize larger cohorts and clinical validation of these biomarkers, as well as exploring their mechanistic roles in disease progression. The integration of these insights into clinical practice may pave the way for improved diagnostic and therapeutic frameworks in AD management.

## 5 Conclusion

This study bridges bibliometric and bioinformatics approaches to elucidate the role of N-glycosylation in AD. Bibliometric analysis highlights a growing focus on molecular mechanisms in AD research, with N-glycosylation emerging as a key area of interest. Bioinformatics identified critical genes such as TMEM59, MLEC, and MAX, which are implicated in glycoprotein homeostasis and neuroinflammatory processes. Machine learning models, particularly logistic regression, demonstrated strong diagnostic potential using minimal gene panels. Additionally, TFs like MAX and BRD9 were linked to glycosylation regulation and glial activation. These findings advance our understanding of N-glycosylation’s role in AD pathogenesis and provide promising targets for early diagnosis and therapeutic intervention.

## Data Availability

The datasets presented in this study can be found in online repositories. The names of the repository/repositories and accession number(s) can be found in this article/Supplementary material.
